# The role and mechanism of NAT10‐mediated ac4C modification in tumor development and progression

**DOI:** 10.1002/mco2.70026

**Published:** 2024-12-04

**Authors:** Zhuoran Gu, Libin Zou, Xinjian Pan, Yang Yu, Yongqiang Liu, Zhijin Zhang, Ji Liu, Shiyu Mao, Junfeng Zhang, Changcheng Guo, Wei Li, Jiang Geng, Wentao Zhang, Xudong Yao, Bing Shen

**Affiliations:** ^1^ Department of Urology Shanghai Tenth People's Hospital School of Medicine Tongji University Shanghai China; ^2^ Urologic Cancer Institute School of Medicine Tongji University Shanghai China; ^3^ Tongji University Cancer Center, Shanghai Tenth People's Hospital, School of Medicine Tongi University Shanahai China

**Keywords:** cancer, epitranscriptome, N4‐acetylation (ac4C), N‐acetyltransferase 10 (NAT10), RNA modification

## Abstract

RNA modification has emerged as a crucial area of research in epigenetics, significantly influencing tumor biology by regulating RNA metabolism. N‐acetyltransferase 10 (NAT10)‐mediated N4‐acetylcytidine (ac4C) modification, the sole known acetylation in eukaryotic RNA, influences cancer pathogenesis and progression. NAT10 is the only writer of ac4C and catalyzes acetyl transfer on targeted RNA, and ac4C helps to improve the stability and translational efficiency of ac4C‐modified RNA. NAT10 is highly expressed and associated with poor prognosis in pan‐cancers. Based on its molecular mechanism and biological functions, ac4C is a central factor in tumorigenesis, tumor progression, drug resistance, and tumor immune escape. Despite the increasing focus on ac4C, the specific regulatory mechanisms of ac4C in cancer remain elusive. The present review thoroughly analyzes the current knowledge on NAT10‐mediated ac4C modification in cancer, highlighting its broad regulatory influence on targeted gene expression and tumor biology. This review also summarizes the limitations and perspectives of current research on NAT10 and ac4C in cancer, to identify new therapeutic targets and advance cancer treatment strategies.

## Graphical abstract

RNA modification is a critical area of study within epigenetics, profoundly influencing the biological functions of tumors. Among these modifications, N4‐acetylcytidine (ac4C) stands out as the sole acetylation modification found in eukaryotic RNA. This modification is catalyzed by N‐acetyltransferase 10 (NAT10). ac4C enhances gene expression by modulating mRNA stability and translation efficiency, thereby playing a pivotal role in various aspects of tumor biology, including tumor progression, drug resistance, and immune evasion.

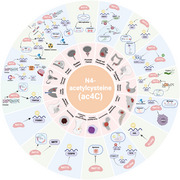

## 1 INTRODUCTION

Classical genetics focuses on alterations in gene function resulting from changes in the genetic sequence, such as mutations, which lead to heritable phenotypic variations. However, epigenetics involves heritable changes in gene function that occur without alternations to the DNA sequence, yet still result in phenotypic changes.^1^, ^2^ This posttranscriptional epigenetic regulation involves various mechanisms, including noncoding RNA (ncRNA) regulation, chromatin remodeling, nucleosome positioning, and RNA modifications, all of which are potential regulators of gene expression.^3^

RNA modification has garnered considerable research attention in recent years. Epigenetic modifications of RNA play a pivotal role in cellular functions by influencing RNA production, transport, metabolism, and translation efficiency, thereby controlling the synthesis of downstream proteins. Common RNA modifications include N6‐methyladenosine (m6A), pseudouridine (Ψ), N1‐methyladenosine (m1A), 5‐methylcytosine (m5C), 7‐methylguanosine (m7G), N4‐acetylcytidine (ac4C),^4^, ^5^ and so on. These modifications have been proved to be pivotal posttranscriptional regulatory mechanisms in physiological and pathological processes. Among these, ac4C is a highly conserved modification widely found in various eukaryotic and prokaryotic RNAs, including transfer RNA (tRNA), ribosome RNA (rRNA), and messenger RNA (mRNA).^6^, ^7^

Hitherto, it was difficult to detect ac4C due to technological limitations. ac4C was first identified in yeast tRNA in 1966 through enzymatic hydrolysis and mass spectrometry (MS) analysis.^8^ ac4C is less abundant than m6A on mRNA. Additionally, ac4C is evolutionarily conserved across diverse species and is catalyzed by N‐acetyltransferase 10 (NAT10) or homologous enzymes in other organisms.^9^, ^10^, ^11^ The cytidine N4 modification does not disrupt cytosine–guanine (C–G) base pairing, rendering it undetectable by standard sequencing or hybridization‐based methods, which contributed to the initial difficulty in detecting ac4C.^12^ To date, NAT10 and its homologs remain the only enzymes known to catalyze ac4C formation on RNA, with no deacetylation enzymes or binding proteins identified. Prior studies primarily focused on ac4C in tRNA and rRNA.^13^ For instance, ac4C facilitated accurate codon recognition on methionine tRNA in *Escherichia coli*,^14^ and was also detected on 18S rRNA in rat hepatocytes.^15^ Recent studies identified significant ac4C modification on mRNA in HeLa cells, revealing that NAT10 plays a pivotal role in boosting mRNA translation efficiency and stability.^16^ Furthermore, ac4C was identified on yeast mRNA, with Rra1, a NAT10 homolog, playing a role in its formation.^17^ Advances in high‐throughput sequencing technologies, along with continuous improvements in bioinformatics tools, have allowed for more detection and quantification of RNA modifications, further promoting ac4C research.

Cancer is the second leading cause of death worldwide, and early diagnosis and development of therapeutic targets are crucial for cancer treatment.^18^, ^19^ Recent studies have shown that the onset and progression of various cancers, including bladder cancer (BCa), breast cancer (BC), cervical cancer (CC), colorectal cancer (CRC), and so on, are associated with NAT10‐catalyzed ac4C modification. Targeting NAT10 has shown significant suppressive effects in various cancers.^19^, ^20^

The present review provides a comprehensive overview of the role and distribution of ac4C in RNA acetylation modification, and the advances in detection tools. The role of NAT10‐mediated ac4C in various tumor progression is also summarized. The review seeks to provide new insights and strategies for the development of innovative targets for cancer therapy and prognosis.

## 2 METHODS FOR DETECTING ac4C

Commonly modified nucleosides produced by RNA hydrolysis can be effectively separated and analyzed using high‐performance liquid chromatography (HPLC). While HPLC is a robust technique for identifying various modified nucleosides in RNA, it demands substantial solvent volumes for effective separation and lacks signal amplification capabilities, which limits its sensitivity.^21^, ^22^, ^23^

Liquid chromatography–mass spectrometry (LC–MS)^24^, ^25^ integrates the capabilities of LC and MS. In this approach, LC facilitates sample ionization and separates the sample based on the mass‐to‐charge ratio of ions, and MS subsequently detects the molecular weight of each ion peak, allowing for detailed sample analysis. LC–MS is known for its precision, sensitivity, and selectivity; however, it requires intricate operational steps and significant pretreatment of RNA samples.^17^

An antibody‐based method employs ac4C‐specific antibodies to enrich modified RNA through immunoprecipitation, followed by identification via deep sequencing. This technique benefits from signal amplification and has been utilized to detect ac4C modification in human and viral mRNA.^16^, ^26^

The borohydride reduction method exploits the chemical characteristic, whereby ac4C‐modified pyrimidine rings exhibit lower electron density compared with cytidine, rendering ac4C reducible by borane. Recently, a method known as ac4C sequencing (ac4C‐seq) has been developed, which uses sodium borohydride under acidic conditions to reduce ac4C to N4‐acetyl‐3,4,5,6‐tetrahydrocytidine. During subsequent reverse transcription, the modified nucleoside is misread as uracil (U) instead of C, causing C‐to‐thymine (C‐to‐T) transitions at ac4C sites in the resulting complementary DNA (cDNA) sequencing.^27^

Computational tools, such as PACES and XG‐ac4C, leverage machine learning algorithms based on XGBoost to predict ac4C modification sites in mRNA.^28^, ^29^

## 3 DISTRIBUTION OF ac4C IN RNAs

**FIGURE 1:**
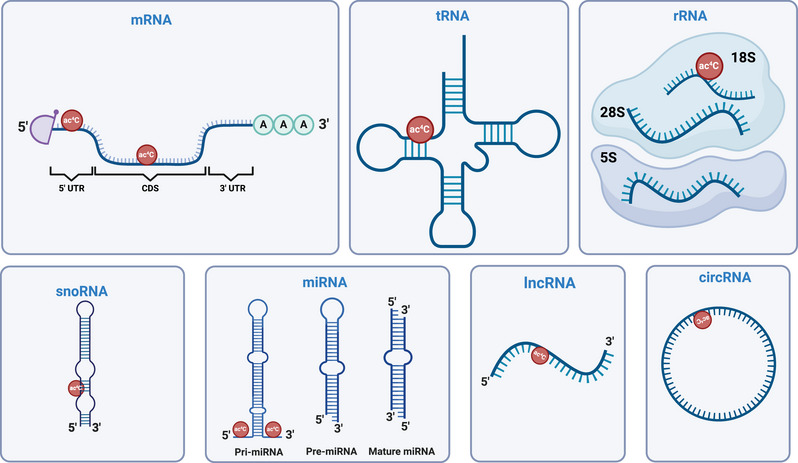
Distribution of ac4C on RNAs. The modification of ac4C occurs on different types of RNAs, including mRNA, tRNA, rRNA, snoRNA, miRNA, lncRNA, and circRNA,. Indicated ac4C modifications are labeled at the corresponding modification sites. ac4C, N4‐acetylcytosine; CDSs, coding sequences; UTR, untranslated regions; snoRNA, small nucleolar RNA; pri‐miRNA primary microRNA, pre‐miRNA precursor microRNA.

ac4C modification is found in various types of RNA, each exerting a distinct functional effect. These RNA types include rRNA, tRNA, mRNA, microRNA (miRNA), and ncRNA (Figure 1).

### 3.1 ac4C on tRNA

In tRNA, ac4C is primarily concentrated at specific sites that significantly influence tRNA stability and functionality.^30^ This modification typically occurs in the anticodon loop and other key structural regions, contributing to the correct folding of tRNA and ensuring accurate codon–anticodon pairing during protein synthesis.^1^, ^31^ ac4C modification was first identified in yeast tRNA^8^ and later in mammals^32^ and at the wobble position of elongator tRNA^Met^ in *E. coli*.^33^ The wobble position refers to the 34th nucleotide in the anticodon loop in the three‐dimensional (3D) structure of RNA, which pairs with mRNA codons and directly influences tRNA recognition specificity and translation efficiency.^34^ ac4C stabilizes the C3′‐endo conformation of ribose, facilitating precise codon readings during translation. This structural stability is vital for maintaining the correct 3D conformation of tRNA, which affects its interaction with mRNA. The formation of ac4C modifications in the D‐loop of tRNA^Ser^ and tRNA^Leu^ is catalyzed by NAT10 with the assistance of thiouridylase methyltransferase and pseudouridine synthase domain‐containing protein 1 (THUMPD1).^17^, ^35^ ac4C is predominantly located at position 12 in eukaryotic tRNA,^36^ and the stability of serine tRNA in Saccharomyces cerevisiae is ac4C dependent.^37^ These findings suggest that ac4C not only influences tRNA folding and structural integrity but also regulates the biosynthesis and degradation of specific tRNA molecules.

### 3.2 ac4C on rRNA

ac4C is primarily situated in the small ribosomal subunit in rRNA. Studies conducted in yeast and human cells have demonstrated that ac4C is located at specific positions within 18S rRNA particularly in the helical regions near the decoding site, which is critical for maintaining translation accuracy. Previous studies based on chemical analysis of rRNA from various species revealed abnormal chemical properties at particular sites, hinting at the presence of previously unidentified modifications.^38^ Subsequent research utilizing more advanced techniques, such as reverse‐phase HPLC (RP‐HPLC) and MS, precisely identified the chemical structure and location of these modifications. ac4C was found to reside in helices 34 and 45 of the 18S rRNA.^34^ Studies explored the role of ac4C in ribosomal function after the determination of these positions.^39^ Comparative studies between wild‐type organisms and ac4C‐deficient mutants showed that ac4C is essential for preserving ribosomal structural stability and ensuring translation. C/D small nucleolar RNA (snoRNA) U13 aid NAT10's binding to 18S rRNA.^40^

### 3.3 ac4C on mRNA

ac4C modifications are predominantly enriched in the coding sequence (CDS) region in mRNA, particularly near the 5′ end, with a decreasing gradient from the 5′ to the 3′ end of the transcript.^16^ This distinct pattern implies a regulatory role of ac4C in mRNA translation initiation and efficiency.^4^ Recent findings have revealed substantial levels of ac4C in both human and yeast mRNA, with a particularly high abundance observed in human HeLa cells. Notably, no cofactors for NAT10 have been identified in the process of ac4C formation in mRNA.

### 3.4 ac4C on miRNA

miRNAs are short single‐stranded RNA molecules that are transcribed by RNA polymerase II or III into primary transcripts (pri‐miRNAs) within the nucleus. These pri‐miRNAs are processed by the DiGeorge syndrome critical region 8 (DGCR8)/drosha ribonuclease III microprocessor complex, exported to the cytoplasm via Exportin‐5, and further cleaved by dicer 1, ribonuclease III.^41^, ^42^, ^43^ The mature miRNA duplex is incorporated into the RNA‐induced silencing complex, where one strand remains to target and silence specific mRNAs.^44^ Recent studies have shown that NAT10/THUMPD1 acetylates pri‐miRNAs with ac4C modifications. These modifications enhance pri‐miRNA interactions with DGCR8, facilitating their processing into precursor miRNAs and promoting the biogenesis of mature miRNAs.^45^

### 3.5 ac4C on ncRNA

Long ncRNAs (lncRNAs) are ncRNA molecules longer than 200 nucleotides, which lack open reading frames^46^ and do not encode proteins. Gene Ontology and Kyoto Encyclopedia of Genes and Genomes pathway analyses have identified 120 ac4C peaks in 102 lncRNAs in Alzheimer's disease (AD),^47^ with 55 showing high acetylation and 47 displaying low acetylation. This suggests that ac4C modification on lncRNAs may be associated with AD pathogenesis.^48^ Further research integrating lncRNA‐seq with nucleotide resolution methods and ac4C–RNA immunoprecipitation revealed that ac4C modification sites in lncRNA CTC‐490G23.2 occur in noncanonical NAT10 motifs, indicating that this lncRNA can be regulated through NAT10‐mediated ac4C modification.^47^

Circular RNAs (circRNAs), generated through back‐splicing of precursor mRNA, exhibit a unique circular structure that makes them resistant to RNA exonucleases resulting in a longer half‐life within cells.^49^, ^50^ circMAST1, a circRNA inhibited the binding of NAT10 to yes‐associated protein (YAP) mRNA in CC, reducing ac4C modification on YAP mRNA.^50^, ^51^ This leads to YAP mRNA degradation, subsequently suppressing tumor growth and metastasis.^52^

## 4 MOLECULAR BIOLOGICAL MECHANISM OF ac4C

ac4C modification occurs specifically at the N4 position of the cytidine base, a nitrogen atom within the C base, which is a pyrimidine present in both RNA and DNA.^53^, ^54^ Typically, the N4 position in the six‐membered ring of C carries an amino group (─NH2), which plays a key role in stabilizing RNA molecules through hydrogen bonding. An acetyl group (─COCH₃) is attached to the N4 nitrogen atom via an amide bond in ac4C modification, forming ac4C. This modification can disrupt the usual C–G base pairing, as the unmodified N4 amino group is involved in standard hydrogen bonding.^3^ Moreover, ac4C decreases the polarity of C and increases its hydrophobicity, influencing the secondary and tertiary structure of RNA, and thereby altering its stability and functionality.^55^

### 4.1 Maintenance of RNA stability

**FIGURE 2:**
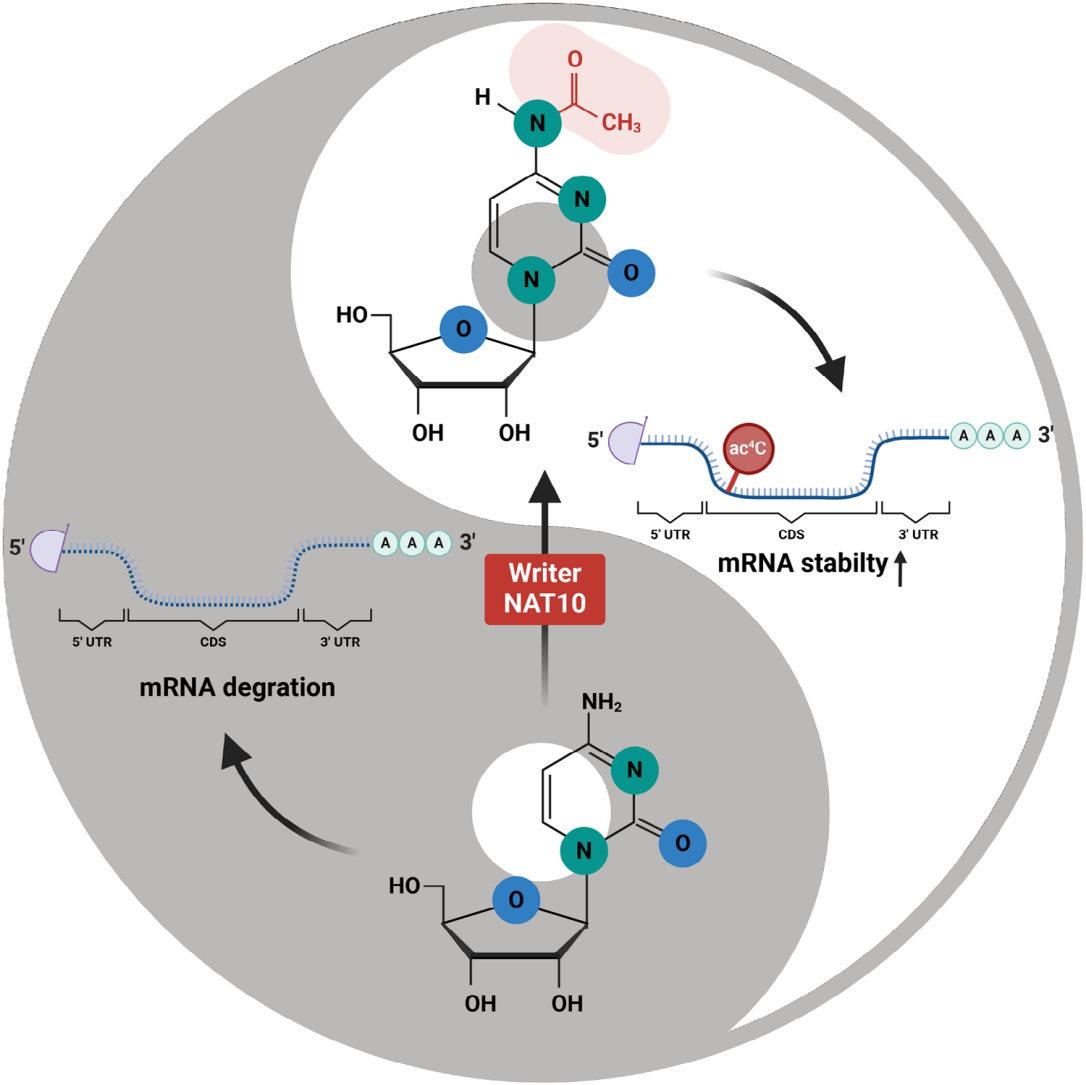
Mechanism and function of ac4C regulated by NAT10 on mRNAs. Mechanism and function of ac4C regulated by NAT10 on mRNAs. Chemical structures of ac4C modifications. ac4C modification can be installed on mRNA, enhances its stability, and thus avoids its fate of degradation. NAT10, N‐acetyltransferase 10; ac4C, N4‐acetylcytosine; CDSs, coding sequences; UTR, untranslated regions.

RNA molecules are typically prone to degradation by ribonucleases. ac4C modification confers structural changes that enhance mRNA resistance to enzyme degradation, thereby prolonging mRNA half‐life. This stabilization is essential for maintaining gene expression and ensuring sustained protein synthesis.^56^, ^57^, ^58^ The specific locations of ac4C modifications on mRNA are instrumental in modulating translation efficiency and stability, as these site‐specific alterations enhance both processes by influencing mRNA conformation and molecular interactions.^16^ ac4C contributes to posttranscriptional gene regulation by stabilizing mRNA, facilitating more efficient recognition by the translation machinery and boosting protein synthesis^6^ (Figure 2).

ac4C modification alters the 3D conformation of RNA, potentially impacting interactions between RNA and various biomolecules,^59^ including proteins, small ligands, and other RNAs. A specific spatial structure is essential for RNA molecules to perform their biological functions, such as regulating mRNA translation or stabilizing tRNA enzymatically.^60^

Key secondary structures, such as hairpins and internal loops, are vital for RNA stability and function, and ac4C plays a critical role in reinforcing these structures, thereby increasing resistance to degradation while maintaining RNA activity. ac4C modifications may occur within CDSs or untranslated regions (UTRs) (such as the 5′ UTR) in mRNA,^61^ where they can regulate translation initiation and affect overall protein production.^62^ ac4C enables cellular adaptation to environmental cues and facilitates the regulation of cell growth, differentiation, and stress responses by modulating mRNA stability and translation efficiency.

### 4.2 Improvement of translational efficiency

ac4C modifications enhance mRNA translation efficiency, improve selectivity and specificity during translation, stabilize mRNA, and extend its half‐life. Furthermore, these modifications can influence the functionality and activity of the resulting proteins.

Typically located in the CDSs or UTRs of mRNA, such as the 5′ UTR, ac4C modifications within the CDSs facilitate translation elongation, whereas modifications in the 5′ UTR inhibit translation initiation by forming inhibitory structures and affecting interactions with tRNA^iMet^. By altering the local secondary structure of mRNA, ac4C enables the translation initiation complex to more easily recognize and bind near the start codon, thus improving the efficiency of translation initiation.^63^ Additionally, ac4C modifications may strengthen interactions between mRNA and ribosomes or other translation factors, further enhancing translation efficiency.^64^ Studies on HeLa cell mRNA have revealed that ac4C significantly affects translation; however, the precise effects vary depending on the location of the modification in the mRNA. ac4C enrichment at wobble positions suggests ac4C role in stabilizing mRNA–ribosome interactions, whereas its presence within the Kozak sequence, a key translation initiation region located upstream of the start codon AUG, has been shown to reduce translation initiation efficiency. Arango et al.^64^ demonstrated that ac4C within the Kozak sequence in HeLa cells decreases translation efficiency, underscoring the nuanced role of ac4C in translation regulation.

ac4C modifications are instrumental in governing the selectivity and specificity of translation for particular mRNAs. These modifications typically occur at specific sites, such as certain regions of the CDSs, within the mRNA, affecting the fidelity of codon recognition during translation. ac4C levels on certain mRNAs may fluctuate under conditions of cellular stress or during development, modulating the translation of these mRNAs to accommodate altered cellular conditions or functional needs.

ac4C modifications in the CDS region stabilize mRNA structure, prolonging the molecule's half‐life and enhancing its cellular persistence and translation potential. This modification aids in resisting degradation by cellular mechanisms, including 5′–3′ exoribonuclease 1 (exonuclease Xrn1), which is responsible for degrading in vitro transcribed reporter constructs.^65^ ac4C contributes to greater mRNA stability and prolonged expression by conferring resistance to Xrn1. The stabilized structure supports higher translation efficiency over extended periods, particularly in cellular states that demand rapid or extensive protein production.

Moreover, ac4C influences the translation of critical regulatory proteins or signaling molecules, thus impacting intracellular signaling pathways and cellular functions. Precise regulation of ac4C modifications can achieve spatiotemporal control of protein expression in specific developmental stages or cell types, facilitating complex biological processes and diverse cellular activities.

## 5 BIOLOGICAL FUNCTIONS OF NAT10

### 5.1 Structure of NAT10

The NAT10 protein is currently recognized as the sole enzyme capable of catalyzing ac4C modifications at multiple RNA sites.^64^ Its acetyltransferase activity regulates mRNA stability and translation efficiency, contributing to various cellular processes, including the modulation of cell death pathways such as apoptosis and autophagy.^66^ NAT10 was initially discovered in 2003 and possesses histone acetylation capabilities. As a member of the G protein subunit alpha transducin superfamily, NAT10 functions as an N‐acetyltransferase, facilitating the acetylation of both histone and nonhistone proteins.^67^, ^68^, ^69^ NAT10 features both an acetyltransferase domain and an RNA‐binding domain, which together enable the catalysis of ac4C modifications across diverse RNA transcripts. During this catalytic process, NAT10 utilizes acetyl‐CoA and adenosine triphosphate, underscoring its integral role in RNA processing and modification, thereby influencing RNA stability and function, and regulating critical cellular processes.^13^, ^65^

NAT10 employs acetyl‐CoA as a substrate to catalyze the acetylation of specific RNA nucleotides, primarily targeting designated sites within tRNA and rRNA, which in turn alters the structure and function of these RNA molecules. Specifically, NAT10 binds to the D‐arm of tRNA, particularly within tRNA^Ser^ and tRNA^Leu^.^16^ THUMPD1, a protein containing a THUMP domain, facilitates NAT10 binding to tRNA, thereby promoting ac4C modifications,^70^ which enhances tRNA stability and translation fidelity. In mammals, NAT10 catalyzes ac4C formation at position 1842 within the terminal helix of 18S rRNA, a vital step in rRNA maturation and ribosome assembly. This modification is critical for the proper folding and maturation of 18S rRNA, as loss of NAT10 results in the accumulation of 18S rRNA precursors and defects in ribosome biogenesis, ultimately impairing cell growth.^40^ This highlights the essential role of NAT10 in regulating cell proliferation and division. Furthermore, NAT10 has been associated with snoRNAs, such as U3 snoRNA, and may contribute to their acetylation, potentially influencing snoRNA functionality and ribosome biogenesis.^69^

Despite these findings, eraser and reader proteins for ac4C remain unidentified. Moreover, the precise molecular mechanisms by which NAT10, the only known ac4C writer, facilitates the incorporation of ac4C residues into RNA continue to be elusive, warranting further research.^71^

### 5.2 ac4C‐dependent functions

NAT10 signaling pathways are involved in multiple cellular processes, including RNA modification, cell cycle regulation, metabolic reprogramming, and cancer development. NAT10 affects mRNA stability and translational efficiency through its ability to catalyze ac4C modification, which in turn regulates multiple signaling pathways. NAT10 regulates solute carrier family 30 member 9 (SLC30A9)’s ac4C by modifying the adenosine monophosphate‐activated protein kinase/mammalian target of rapamycin signaling and promotes tumorigenesis in diffuse large B‐cell lymphoma.^72^ The positive feedback loop of the NAT10/MYB binding protein 1a (Mybbp1a)/p53 axis promotes iron death in cardiomyocytes, and NAT10 induces ac4C modification of Mybbp1a, which increases its stability, leading to the activation of p53 and the subsequent repression of the transcription of the iron‐death‐resistant gene, SLC7A11, which exacerbates cardiac ischemia–reperfusion (I/R) injury.^73^ RN1a is a serine structural domain that is not involved in cardiac I/R injury. Serine structural domain 1 stabilizes NAT10 protein and promotes translational 4C modification in cancer via tRNA‐ac.^74^ NAT10 promotes CRC progression through the NAT10/kinesin family member 2A (KIF23)/glycogen synthase kinase 3 beta (GSK‐3β)/β‐catenin axis, and its expression is mediated by GSK‐3β in the feedback loop.^75^ NAT10 regulates the vascular endothelial growth factor A‐mediated phosphatidylinositol 3‐kinase/protein kinase B signaling pathway through ac4C modification and promotes osteogenic differentiation of periodontal ligament stem cells.^76^ α‐Microtubulin acetyltransferase 1 regulates the activity of NAT10 through lactonization, which is facilitated by the Kaposi's sarcoma‐associated herpesvirus‐encoded polyadenylate ribonucleic RNA that enhances the ac4C modification of the tRNA by NAT10, which in turn facilitates the reactivation of the Kaposi's sarcoma‐associated herpesvirus from latency and enhances the virus's ability to translate and replicate.^77^

#### 5.2.1 Regulation of cell cycle

During meiosis in male germ cells, prophase I of meiosis involves complex cell cycle regulation, including repair of DNA double‐strand breaks (DSBs) and homologous chromosome associations.^7^ NAT10 is localized in the nucleus of male germ cells and ac4C modification is tightly regulated, which exhibits dynamic changes during spermatogenesis.^78^, ^79^ NAT10 deletion results in many dysregulated transcripts. This severely affects spermatogonial differentiation and meiotic entry and leads to defects in synaptonemal complex assembly, homologous recombination and DSB repair, ultimately leading to male sterility.^7^ Male germ cells are unable to complete homologous chromosome pairing, association, and recombination in the absence of NAT10, leading to meiotic arrest. ac4C reduces meiotic defects in spermatozoa.^7^ In addition, ac4C promotes osteoblast differentiation. NAT10 upregulation altered human and mouse mesenchymal stem cells (MSC) mRNAs, promoting osteogenic differentiation of human MSCs and regeneration of mouse corneal cells.^80^, ^81^, ^82^ Human and mouse MSC mRNAs were modified. This suggests that despite the lower abundance of mRNA modifications compared with DNA and histone modifications, it still plays a unique physiological role in male germ cell development.^83^

NAT10 can mediate prostate cancer (PCa) cell proliferation through high mobility group AT‐hook 1 (HMGA1)‐mediated cell cycle alterations.^84^ NAT10 knockdown significantly reduced both ac4C modifications of HMGA1 and mRNA stability in cells, leading to a decrease in mRNA and protein abundance, resulting in a significant G1/S phase block in PCa cells, and the G1/S phase checkpoint is critical for cell proliferation.^78^, ^79^, ^85^ It was shown that p53 acts as a transcription factor that activates downstream p21 transcription and inhibits several cell cycle protein‐dependent kinases, thereby blocking cell cycle G1/S phase progression.^83^, ^86^, ^87^ Similar to p21, p27 is a member of the cell cycle protein‐dependent kinase protein family that binds to several cell cycle protein complexes, thereby inhibiting their function and enhancing the G1/S detection point of the cell cycle.^87^, ^88^, ^89^, ^90^ NAT10 knockdown significantly upregulated protein expression of p27 and p21, and NAT10 promoted cell cycle progression in PCa cells.

#### 5.2.2 Regulation of metabolic reprogramming

NAT10 modulates the mRNA stability and translational efficiency of key enzymes and regulators in several metabolic pathways by catalyzing the formation of ac4C, thereby realizing cellular metabolic reprogramming.^87^, ^88^ Specifically, NAT10 modified the mRNAs of key glycolytic enzymes and regulatory factors during glycolysis and energy metabolism via ac4C to enhance their stability and promote intracellular sugar metabolism and lactate production. For instance, NAT10 modifies the mRNAs of fatty acid metabolism‐related genes through ac4C to enhance their stability and regulate fatty acid synthesis and catabolism processes.^91^, ^92^ This mechanism plays an important role in cellular energy metabolism, especially in maintaining cell membrane composition and adapting to environmental changes.^93^ NAT10 upregulates the transcription factor Forkhead box P1 (FOXP1) in CC to form the NAT10/ac4C/FOXP1 axis, which accelerates glycolytic metabolism, increases lactate production, and promotes immunosuppression in the tumor microenvironment by reprogramming glycolysis.^94^ NAT10 is also delivered to macrophages in the tumor microenvironment via exosomal packaging, promoting macrophage M2‐type polarization and biasing lipid metabolism, further supporting immune escape from tumors.^95^ ac4C enhances the stability of mRNAs encoding enzymes involved in energy production, such as key enzymes in glycolysis, under conditions of high energy demand or limited energy supply. This helps maintain cellular energy homeostasis and ensures cell survival and function during energy deprivation periods.^96^, ^97^, ^98^ In conclusion, NAT10 regulates gene expression and translation of key metabolic pathways, such as glycolysis, fatty acid metabolism, and amino acid metabolism, through ac4C modification, thus playing an important role in energy production, material synthesis, and immune regulation in the tumor microenvironment.

#### 5.2.3 Regulation of immune microenvironment

In adapting to the cellular microenvironment, ac4C regulates mRNA stability and translation, allowing the cell to adapt to changes in external signals such as nutrient supply, oxygen levels, and the extracellular matrix state. Cells need to alter their metabolism in response to inadequate oxygen supply under hypoxic (deficient oxygen) conditions.^99^, ^100^ ac4C regulates the stability and translation of mRNAs encoding inflammatory mediators such as cytokines and chemokines in inflammatory environments, thereby affecting the intensity and duration of the inflammatory response. ac4C may help cells adjust their behavior in inflammatory and immune responses by regulating the production of these mediators. Studies reported a positive correlation between the expression level of NAT10 and the infiltration of M0‐type macrophages. In addition, CD4^+^ T cells are activated when exposed to external antigens and play a central role in the immune system, rapidly releasing large amounts of cytokines.^101^, ^102^ NAT10 enhances programmed cell death‐ligand 1 (PD‐L1) expression by acetylating nucleophosmin 1, which allows tumor cells to evade immune surveillance more effectively. NAT10 inhibitors reduces PD‐L1 expression, thereby enhancing the efficacy of immune checkpoint inhibitors.^103^ NAT10 enhances the stability of polyadenylated nuclear (PAN) RNA through ac4C modification, which supports viral resuscitation and promotes viral gene expression. Meanwhile, ac4C‐modified PAN RNA activates interferon‐gamma inducible factor 16‐associated inflammasomes and enhances immune system response to viruses and tumors. This process directly affects the tumor immune microenvironment and enhances host immune surveillance and attack against tumors^49^ NAT10 enhances mRNA translation of tumor‐associated inflammatory signaling pathways such as interleukin‐6 (IL‐6) and IL‐8 by mediating ac4C modification. This enhanced translation supports an immunosuppressive environment in tumors and helps tumor cells evade immune surveillance.^74^

### 5.3 ac4C independent functions

The non‐ac4C functions of NAT10 are those that are not exerted via the classical ac4C modifying actions. These functions are independent of its RNA acetylation activity and involve diverse cellular processes, especially DNA repair, cytoskeleton regulation, and epigenetics. Evidence indicates that NAT10 promotes oocyte maturation, increases the dynamic complexity of microtubules, and supports asymmetric division of mammalian oocytes during cell growth and development.^104^, ^105^, ^106^ The lack of NAT10 is a major contributor to oocyte maturation in mice. In mice, lack of kinesin family member C1 protein affects actin distribution and NAT10‐catalyzed microtubule acetylation, leading to first polar body efflux failure. In addition, NAT10 regulates the stability of mRNAs of antioxidant enzymes such as superoxide dismutase^107^ and catalase,^108^ thereby facilitating the capability of cells to cope with oxidate stress. During oxidative stress, overexpression of these enzymes decreases the reactive oxygen species‐induced damage^109^, ^110^ and confers protection against oxidative stress.^109^ The C‐terminal intrinsically disrupt the region of NAT10 to alter the liquid–liquid phase separation (LLPS) and regulate m6A reader YTH N6‐methyladenosine RNA binding protein 1 (YTHDF1) splicing thereby promoting gastric cancer (GC) progression.^111^ Serine and arginine rich splicing factor 2 (SRSF2), a splicing factor acetylated by NAT10, was reported to bind directly to the pre‐mRNA of the m6A reader YTHDF1, enhancing the YTHDF1 exon 4 skipping and upregulating the short YTHDF1 transcript, which stimulates the proliferation and migration of GC cells.^111^

## 6 OVERVIEWS OF NAT10 FUNCTIONS IN CANCER

**FIGURE 3:**
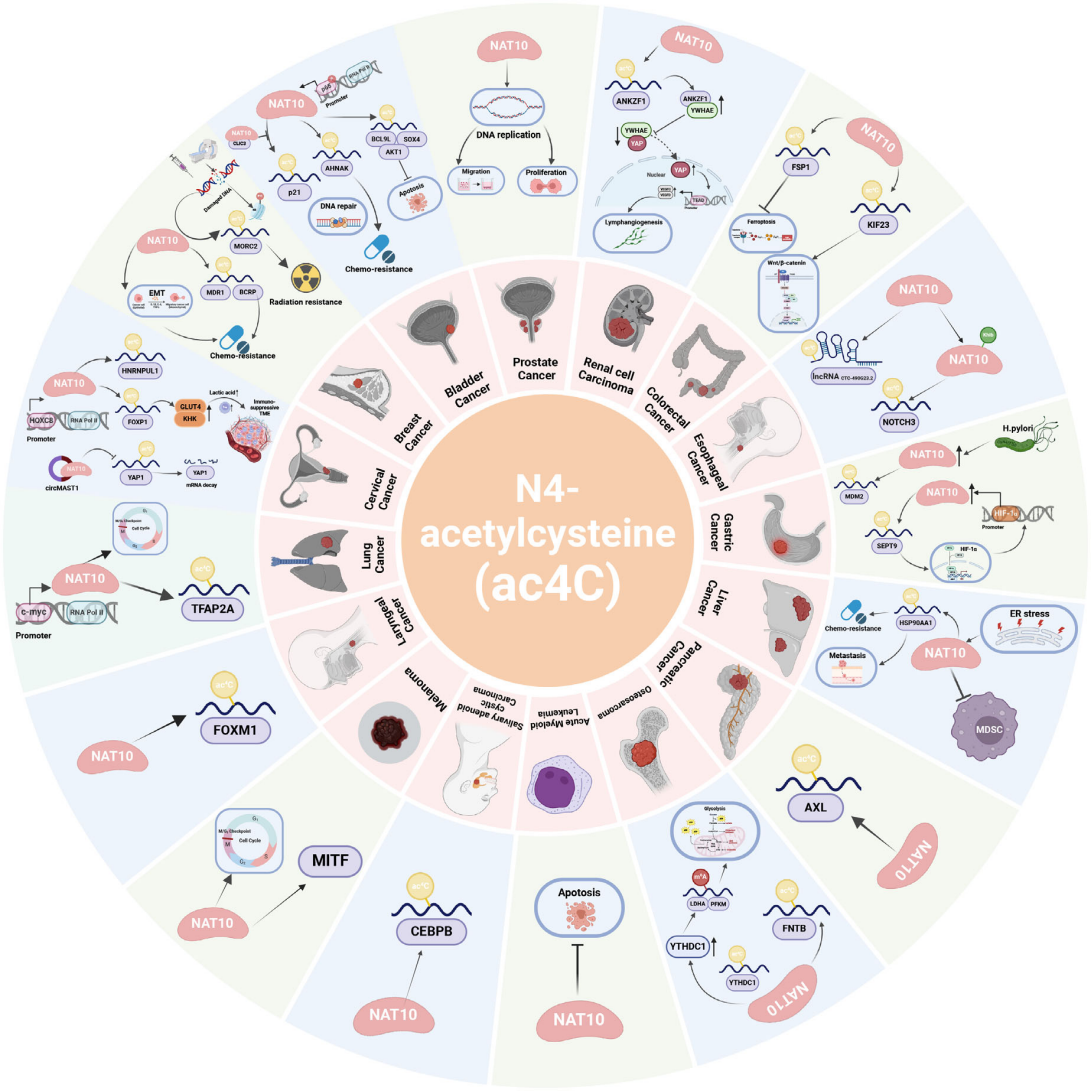
NAT10 plays a critical role in cancer. ac4C plays a positive role in regulating the progression of various cancers as illustrated. In bladder cancer, NAT10 is activated by NF‐κB signaling, promotes the expression of BCL9L, SOX4, AKT1 through ac4C to inhibit apoptosis of cancer cells. It also mediated DNA damage repair and chemotherapy resistance through ac4C modification of AHNAK and p21. In breast cancer, NAT10 promoted tumor chemoresistance by regulating the activation of EMT and ac4C modification of MDR1 and BCRP. Radiotherapy‐ and chemotherapy‐mediated DNA damage activates the ac4C modification of MORC2 to regulate the cell cycle, enhancing treatment resistance. In cervical cancer, NAT10 is transcriptionally activated by HOXC8, and it regulates the expression of GLUT4 and KHK by enhancing the ac4C modification of FOXP1, thus promoting tumor lactate production and the development of immunosuppressive microenvironment. In addition, NAT10 can be suppressed by circMAST1, affecting the stability of the critical transcription factor YAP1. In colorectal cancer, upregulation of NAT10 promotes ac4C modification of FSP1 to facilitate ferroptosis resistance and stimulates WNT/β‐catenin pathway via KIF23. In various tumors, NAT10 mediates tumor progression, metastasis, therapy resistance, and the formation of immunosuppressive microenvironment by promoting ac4C activation of downstream oncogenic pathways. Khib, lysine 2‐hydroxyisobutyrylation; MITF, microphthalmia‐associated transcription factor; EMT, epithelial–mesenchymal transition; ER stress, endoplasmic reticulum stress; MDSC, myeloid‐derived suppressor cell; ANKZF1, ankyrin repeat and zinc finger peptidyl TRNA hydrolase 1; YWHAE, tyrosine 3‐monooxygenase/tryptophan 5‐monooxygenase; FSP1, ferroptosis suppressor protein 1; KIF23, kinesin family member 23; NOTCH3, Notch receptor 3; MDM2, the murine double minute 2; SEPT9, septin 9; AXL, AXL receptor tyrosine kinase; FNTB, the farnesyltransferase subunit beta gene; YTHDC1, YTH N6‐methYLADENOSINE RNA BINDING PROTEIN C1; m6A, N(6)‐methyladenosine; CEBPB, CCAAT enhancer binding protein beta; FOXM1, Forkhead box M1; TFAP2A, transcription factor AP‐2 alpha; HNRNPUL1, heterogeneous nuclear ribonucleoprotein U like 1; FOXP1, Forkhead box P1; GLUT4, glucose transporter 4; KHK, ketohexokinase; MORC2, MORC family CW‐type zinc finger 2; MDR1, breast cancer resistance protein 1; BCRP, breast cancer resistance protein; BCL9L, B‐cell lymphoma 9‐like.

**FIGURE 4:**
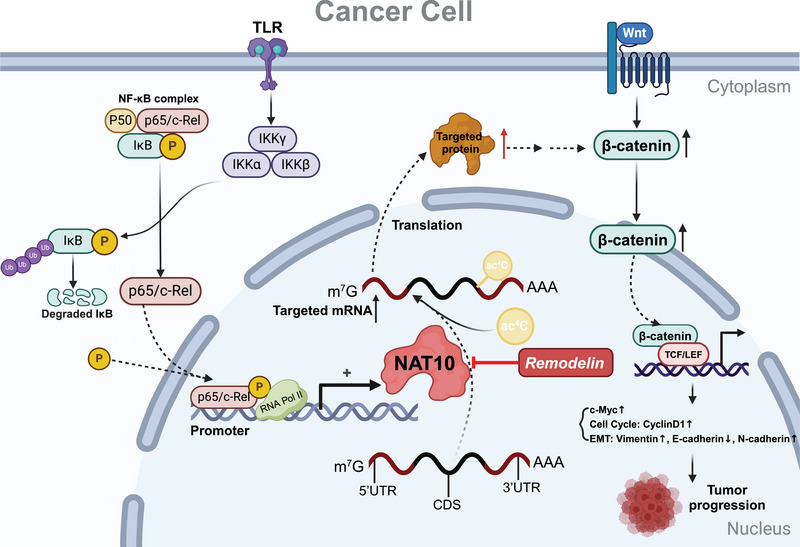
Overview of the ac4C‐related signaling pathway in cancer. In cancer cells, activation of the NF‐κB signaling pathway promotes NAT10 expression. Activated p65 binds to the NAT10 promoter and regulates its expression in a transcriptional manner. NAT10, inhibited by Remodelin, mediates mRNA ac4C modification and promotes the downstream protein expression thereby activating the Wnt/β‐catenin pathway. NAT10 boosts c‐Myc upregulation, regulates cell cycle and EMT through activation of the Wnt/β‐catenin pathway, ultimately promoting tumor progression. NF‐κB, nuclear factor kappa‐B; TCF/LEF, T‐cell factor/lymphoid enhancer‐binding factor. TLR, Toll‐like receptor; EMT, epithelial–mesenchymal transition.

Evidence from recent studies has demonstrated that NAT10 and its associated ac4C modification promote the tumor progression of multiple cancer types. In the following sections, we explore the specific mechanisms by which NAT10 and ac4C modifications contribute to cancer advancement in different cancer malignancies (Figure 3). Besides, NAT10 can regulate the signaling pathways in cancer, which influences proliferation, migration, and apoptosis of cancer cells. Figure 4 illustrates the intricate network of the signaling pathways modulated by NAT10 in pan‐cancer, highlighting its central role in tumor progression (Figure 4).

### 6.1 Acute myeloid leukemia

NAT10 regulates the malignant proliferation of acute myeloid leukemia (AML) cells by targeting the cell cycle and inhibiting apoptosis. Treatment with Remodelin, a NAT10 inhibitor, enhanced endoplasmic reticulum stress (ERS), as indicated by the increased GRP78 and caspase‐12 cleavage, and UPR activation via upregulation of IRE1, CHOP, and PERK. Remodelin was found to trigger the classical apoptotic pathway by upregulating Bax/Bak and downregulating Bcl‐2.^112^

### 6.2 Bladder cancer

NAT10 is overexpressed in BCa and correlated with poor patient prognosis. It contributes to cisplatin resistance by mediating DNA damage repair (DDR) via the stabilization of specific mRNAs. Moreover, acetylation of NAT10 influence cell cycle regulation and tumor proliferation. Targeting NAT10 represents a promising therapeutic approach for overcoming cisplatin resistance and tumor growth of BCa.

Elevated NAT10 levels enhanced the proliferation, migration, stemness, and reduce apoptosis in BCa cells by increasing the ac4C acetylation of critical downstream mRNAs, including BCL9L, SOX4, and AKT1. This modification improves the stability and translation efficiency of these mRNAs, thereby advancing the malignant features of BCa cells^113^ Lin et al.^114^ further demonstrated that NAT10 exacerbates cisplatin resistance in BCa by increasing DDR. Treatment with cisplatin activated the NF‐κB signaling pathway in BCa cells, promoting the p65 binding at the NAT10 promoter region, to enhance gene transcription. Overexpression of NAT10 promotes the ac4C acetylation of AHNAK mRNA, stabilizing it against exonuclease‐mediated degradation. In this context, NAT10‐induced DDR is mediated by AHNAK, leading to cisplatin resistance. Importantly, targeted inhibition of NAT10 by Remodelin can inhibit BCa progression and reverse cisplatin resistance in vitro and in vivo. These findings suggest that NAT10 may be a potential therapeutic target for preventing cisplatin resistance, and its inhibition can restore cisplatin sensitivity in BCa.

Furthermore, the interaction between chloride intracellular channel 3 (CLIC3) and NAT10 had been reported, in which CLIC3 inhibits the acetylation of NAT10, thereby reducing ac4C modification levels on p21 mRNA.^115^ As a member of the cyclin‐dependent kinase (CDK) inhibitor family, p21 can bind and inhibit CDKs, which suppresses the DNA damage‐induced p53‐mediated cell cycle progression and suppressing tumor proliferation.^116^

### 6.3 Breast cancer

The treatment options for BC include surgery and adjuvant therapies, such as endocrine therapy, chemotherapy, and radiotherapy. BC patients showing the resistance to adjuvant therapy, exhibit poor survival and prognosis. In BC, ac4C mediated by NAT10 regulates the occurrence of resistance to chemotherapy and radiotherapy suggesting that the writer NAT10 is a potential therapeutic target for overcoming resistance to clinical treatment.

NAT10 stimulates the ac4C modification of key drug resistance genes, including breast cancer resistance protein 1 and breast cancer resistance protein in BC. A previous study indicated that inhibition of NAT10 using Remodelin effectively restored drug sensitivity in capecitabine‐resistant BC cell lines, underscoring the critical role of NAT10 in BC chemoresistance.^117^ Li and colleagues^54^ further revealed that, in BC, DNA damage induced by radiotherapy and chemotherapy increases the acetylation of MORC family CW‐type zinc finger 2 (MORC2) mediated by NAT10, particularly at lysine 767. This acetylation enhances the binding of MROC2 to histone H3 phosphorylated at threonine 11, a modification induced by DNA damage. This interaction inhibits the transcription of CDK1 and cyclin B1,^118^, ^119^ activating the G2 checkpoint in response to DNA damage, enhancing tolerance to radiotherapy and chemotherapy. This suggests that targeting NAT10 may be a therapeutic strategy for overcoming resistance to these treatments in BC.^120^ In addition, NAT10 has been shown to induce doxorubicin resistance in BC by facilitating epithelial–mesenchymal transition (EMT).^121^ However, the study did not perform in vivo and in vitro phenotypic experiments, and hence, the role of NAT10‐mediated ac4C modification in doxorubicin resistance needs to be clarified.^122^

### 6.4 Cervical cancer

NAT10 is an oncogene involved in CC and facilitates ac4C modification of targeted RNAs in CC, influencing glycolysis and the immune microenvironment.^123^ Additionally, NAT10 serves as an RNA‐binding protein, which is regulated by specific circRNA.^124^

In CC, HOXC8 binds to the promoter region of NAT10, upregulating its expression in cancer tissues. NAT10, in turn, mediates the ac4C acetylation of FOXP1 mRNA, enhancing FOXP1 protein expression. This upregulation promotes the expression of key glycolytic enzymes, including GLUT4 and KHK, enhancing glycolysis and lactate secretion in CC cells. These metabolic shifts creates a lactate‐rich inhibitory immune microenvironment by activating tumor‐infiltrating regulatory T cells.^94^ In vivo experiments further demonstrated that NAT10 knockout improves the inhibitory effect of PD‐L1 on tumors. Activation of the NAT10/FOXP1 axis in CC cells inhibits immune response by modulating glycolysis in tumor cells. Consequently, targeting NAT10 and PD‐1/PD‐L1 inhibitors are promising strategies for enhancing CC immunotherapy. This study demonstrates a link between tumor lactate metabolism and the immune microenvironment via NAT10‐mediated ac4C modification. The expression of NAT10 in CC was found to be increased in TCGA and GEO databases through bioinformatics analyses. Using acRIP‐seq, researchers identified that NAT10 mediates the ac4C modification of HNRNPUL1 mRNA, stabilizing it. Furthermore, knockdown of HNRNPUL1 reverses the oncogenic phenotype induced by NAT10.^125^ Yao et al.^52^ investigated the role of circRNAs in CC and found that circMAST1 is expressed at low levels in tumor tissues, correlating with poor prognosis. They found that circMAST1 competitively binds to NAT10, disrupting its interaction with YAP mRNA. This interference reduces the ac4C modification of YAP mRNA, causing its degradation and inhibition of tumor progression.^52^

### 6.5 Colorectal cancer

NAT10 is overexpressed in CRC tissue and ac4C modification of targeted genes mediated by NAT10 inhibits ferroptosis and activates Wnt/β‐catenin pathway to promote the progression of CRC.

NAT10 is overexpressed in CRC, binds to the 3′ UTR of KIF23 mRNA, and catalyzes its ac4C modification. This acetylation enhances KIF23 translation, inducing Wnt/β‐catenin pathway activation to promote CRC progression. In vivo and in vitro experiments have unconvered that inhibiting NAT10 expression by Remodelin reversed NAT10‐induced functional phenotypes, including cell proliferation, migration, invasion, G2/M cell cycle regulation, liver and lung metastasis, and inhibition of apoptosis, all of which are linked to tumor progression.^75^ NAT10 mediates the ac4C modification of ferroptosis suppressor protein 1 mRNA, thereby inhibiting ferroptosis of CRC cells, facilitating tumor progression.^126^ Liu et al.^127^ established a risk regression model for RNA acetylation based on acetylation‐related gene sets, which allowed the calculation of the acetylation risk scores for patients with CRC undergoing immunotherapy based on the public databases. A positive correlation was observed between acetylation risk scores and key factors such as tumor mutation burden, immune cell infiltration, microsatellite instability and patient responsiveness to immunotherapy. However, the study was based on bioinformatics analysis. Therefore, these findings should be clarified through in vitro and in vivo experiments.^127^ Zhang et al.^128^ identified the subcellular relocalization of NAT10 in CRC, demonstrate its translocation from the nucleus to the cell membrane. This relocalization, triggered by the inhibition of GSK‐3β expression, caused alterations to the cytoskeletal dynamics, which enhanced the tumor cell motility and CRC progression.^128^

### 6.6 Esophageal cancer

The oncogenic mechanism of NAT10 in esophageal cancer (ESCA) has been extensively investigated. NAT10's lysine 2‐hydroxyisobutyrylation (Khib) modification at lysine 823 activates the NOTCH3 signaling pathway, driving metastasis of ESCA. Furthermore, NAT10 can mediate ac4C modifications in tRNA and lncRNA.

Liao et al.^129^ uncovered a novel posttranslational modification, Khib, that was markedly overexpressed in primary and metastatic ESCA. Notably, the amino acid 823 was found to be a critical site for Khib modification of NAT10, linking this modification to poor prognosis. NAT10 K823‐Khib modification was enhanced during tumor metastasis, demonstrating its involvement in tumor biology. To investigate the functional implications of NAT10 K823‐Khib, a NAT10 K823R mutant was designed to simulate a non‐Khib modification state. Through comprehensive knockout and replication experiments conducted both in vivo and in vitro, it was found that the K823R mutant failed to replicate the tumor‐suppressing effects observed after NAT10 knockout, indicating that Khib modification is essential for the NAT10's pro‐metastatic activity. Further investigations into the downstream mechanisms identified the ac4C modification checkpoint on NOTCH mRNA, which was validated using a single base chemical sequencing strategy. Collectively, the results confirmed the presence of ac4C modification on NOTCH3 mRNA molecules at the single‐base level, uncovering a signaling cascade involving NAT10 (K823 Khib) and NOTCH3 (ac4C), which regulates fibronectin during tumor metastasis. The identification of mRNA ac4C modification provided direct evidence of its regulatory function. Moreover, the authors reported an important compound that inhibits NAT10 K823‐Khib modification to inhibit lung cancer development, providing a new avenue for antitumor drug development.^129^

Elsewhere Lin et al.^130^ reported that NAT10 promotes the ac4C modification of tRNA to enhance the translation efficiency of EGFR mRNA by optimizing tRNA decoding at specific codons. They also provided the first evidence that combining NAT10 inhibition with gefitinib effectively suppressed ESCA progression, as validated through in vivo and in vitro experiments.^130^

Although ac4C modification is well known to enhance mRNA stability and translation efficiency, its role in on noncoding RNAs remains largely unexplored. Li's pioneering research illuminated the role of ac4C modification in lncRNAs, particularly in esophageal squamous cell carcinoma (ESCC). Their findings revealed that NAT10 mediates ac4C modification of lncRNAs, notably CTC‐490G23.2, in both primary and metastatic ESCC. This modification stabilizes and upregulates CTC‐490G23.2 which in turn, promotes cancer invasion and metastasis by regulating the splice‐switching of CD44 precursor mRNA, leading to an oncogenic isoform that enhances vimentin stability. Overexpressed of CTC‐490G23.2 and its CD44 isoform was linked to poor prognosis. Additionally, antisense oligonucleotide nanocomplexes targeting CTC‐490G23.2 have shown potential in inhibiting metastasis, offering a promising therapeutic strategy for improving outcomes in ESCA.^47^

### 6.7 Gastric cancer

Hypoxia is a common feature of solid tumors microenvironmental. The study by Li et al.^131^ reported increased ac4C modification levels in GC cells under hypoxic conditions. They also found that the writer protein NAT10 activated the HIF‐1 pathway by modulating the acetylation of SEPT9. Concurrently, the transcription factor HIF‐1α enhanced NAT10 transcription, creating a positive feedback loop that sustains HIF‐1 pathway activation and promotes glycolysis in GC cells. In addition, it was observed that inhibiting the NAT10 expression with Remodelin, in combination with antiangiogenic therapy (Apatinib), successfully decreased tumor progression in in vivo and in vitro.^131^

Microorganisms have been reported to participate in ac4C modification, suggesting that they may influence GC progression by modulating ac4C. Helicobacter pylori, a major pathogenic factor in GC, increases NAT10 expression in GC tissues. NAT10 can modulate the ac4C acetylation of MDM2, enhancing MDM2 expression and decreasing the expression level of tumor suppressor gene p53. This molecular cascade drives GC progression.^59^ These findings revealed the mechanistic relationship between oncogenic microorganisms and RNA modification.

Neutrophil extracellular traps (NETs), web‐like structures composed of DNA, histones, elastin, and antimicrobial peptides, influence the occurrence of infections, autoimmune diseases, and cancer. In the tumor microenvironment, NETs recruit neutrophils, which promote tumor growth. Moreover, NETs increase the risk of tumor recurrence by reactivating dormant tumor cells. Wang et al.’s^133^ research suggests that NETs stimulated GC cell proliferation and metastasis via NAT10‐mediated ac4C acetylation of MYND^132^ domain containing 2.

LncRNA DARS‐AS1 was reported to be overexpressed in GC.^134^, ^135^ DARS‐AS1 acts as a molecular sponge, sequestering miR‐330‐3p, thereby downregulating NAT10 expression. This interaction forms a competing endogenous RNA (ceRNA) network, specifically lncDARS‐AS1/miR‐330‐3p/NAT10, that promotes GC progression.^134^ However, conflicting data from other studies showing increased NAT10 expression in GC tissues, and the lack of in vivo functional assays indicates that the role of this regulatory network in GC need to be further explored.

Liu et al.^137^ further showed that NAT10 undergoes LLPS mediated by the intrinsically disordered region at the C‐terminal domain of NAT10. Furthermore, NAT10 interacts with SRSF2 and mediates the acetylation of SRSF2 at lysine 52 (K52), thereby enhancing the stability of the SRSF2 protein. SRSF2 regulates the alternative splicing of YTHDF1, accelerating the progression of GC.^137^

In GC, NAT10 mediates ac4C modification of targeted mRNAs to activate signaling pathways and also undergoes LLPS and mediates the acetylation of interacted proteins to improve their stability.

### 6.8 Laryngeal cancer

In laryngeal cancer, NAT10 is highly expressed in which promotes the progression of the disease by mediating ac4C acetylation of the downstream target gene FOXM1.^138^

### 6.9 Lung cancer

Immunohistochemistry analysis of tissue microarrays identified significantly higher levels of ac4C modification and its key enzyme NAT10 in lung adenocarcinoma tissues compared with adjacent normal tissues. Further bioinformatics analysis and acRIP‐qPCR confirmed the presence of ac4C acetylation checkpoints in the downstream gene TFAP2A, underscoring the role of NAT10 in lung adenocarcinoma progression.^139^ Wang et al.^140^ analyzed non‐small cell lung cancer (NSCLC) samples from public databases and their own research cohorts. They observed that NAT10 expression was upregulated. Chromatin immunoprecipitation assays identified the transcription factor c‐Myc as a key regulator of NAT10 transcription. Pathway analyses and Western blotting further confirmed that NAT10 modulates NSCLC cell proliferation and invasion by modulating the cell cycle and causing G1/M phase arrest. However, this study have not been confirmed through in vivo validation, and the expression patterns and functions of NAT10 across different lung cancer subtypes need to be investigated.^140^

### 6.10 Liver cancer

NAT10‐mediated ac4C modification influences the tumor microenvironment and immune cell function in HCC. Additionally, NAT10 stabilizes downstream targeted mRNAs, which promote metastasis and resistance to HCC apoptosis.

NAT10 is highly expressed in hepatocellular carcinoma (HCC) cell lines. Knockdown of NAT10 using siRNA or inhibition via Remodelin decreased EMT by downregulating E‐cadherin and upregulating vimentin, which prevented the proliferation and invasion of HCC cells.^141^ In addition, ac4C acetylation was found to affect immune cell the infiltration and function within the tumor microenvironment, as evidence by orthotopic liver cancer mouse models and single‐cell RNA‐seq analyses. Inhibiting of ac4C modification using Remodelin increased the infiltration of myeloid‐derived suppressor cells (MDSCs) and upregulated PD‐L1 expression on these cells. This heightened PD‐L1 expression subsequently impaired cytotoxic T lymphocyte‐mediated tumor cell killing. Therefore, targeting ac4C modification may enhance the effectiveness of immunotherapy for liver cancer by downregulating PD‐L1 expression on MDSCs.^142^

ERS occurs when the functionality of the endoplasmic reticulum (ER) is disrupted, typically due to excessive protein synthesis, protein misfolding, or environmental changes. In response, cells activate the unfolded protein response (UPR), which aims to restore ER function by reducing protein synthesis, improving protein folding capacity, and promoting the degradation of misfolded proteins. If ER stress persists and the UPR cannot re‐establish cellular equilibrium, apoptosis may be triggered. Therefore, ERS is considered as an important factor influencing tumor progression, and manipulating this stress response to induce cancer cell death or increase chemotherapeutic sensitivity is increasingly be explored. Studies have shown that NAT10 exacerbates the metastatic potential and resistance to apoptosis of HCC cells under ER stress conditions. By upregulating ac4C modification of HSP90AA1, NAT10 stabilizes its mRNA and enhances HSP90AA1 expression. Increased HSP90AA1 levels were reported to promote metastasis and increase the resistance of HCC cells to lenvatinib‐induced apoptosis, highlighting the pivotal role of NAT10 in modulating ERS‐mediated HCC progression.^143^

### 6.11 Malignant melanoma

NAT10 was found to promote malignant melanoma progression by regulating the cell cycle and microphthalmia‐associated transcription factor, further underscoring its role in various malignancies.^144^

### 6.12 Osteosarcoma

Available evidence demonstrates the increased ac4C modification levels and expression of the writer protein NAT10 in osteosarcoma (OS). NAT10, through its ac4C modification activity, upregulates the expression of the downstream m6A reader protein YTHDC1, which recognizes the m6A modification sites in critical glycolysis enzymes, such as phosphofructokinase and lactate dehydrogenase A. This recognition boosts their translation efficiency, driving OS progression by enhancing glycolysis.^145^ Other studies have reported that NAT10 mediates the ac4C modification of the farnesyltransferase subunit beta gene, further contributing to the progression of OS.^146^ Fan et al.^147^ explored the therapeutic potential of Remodelin to inhibit NAT10 expression in OS cell lines. Through acRIP‐seq and network pharmacology analyses of mRNAs with reduced ac4C modification, along with functional validation in vitro, ESR2, IGF1, and MAPK1 were identified as key molecular targets involved in Remodelin's suppression of the oncogenic phenotype in OS.^147^

### 6.13 Pancreatic cancer

Elevated NAT10 expression in pancreatic ductal adenocarcinoma (PDAC) increases the stability and enhances the expression of AXL mRNA through ac4C modification, thereby promoting pancreatic cancer progression. Furthermore, a predictive model for clinical outcomes in patient with PDAC was developed based on transcriptome data from the TCGA and GEO datasets, focusing on ac4C‐related signatures.^148^

### 6.14 Prostate cancer

Remodelin is able to inhibit NAT10 expression in both hormone‐sensitive and castration‐resistant PCa cell lines.^84^, ^149^ In vivo and in vitro experiments showed significant reductions in PCa cell proliferation, migration, and invasion.^61^ DNA fiber assays revealed concurrent inhibition of DNA replication forks, with NAT10 showing consistent expression patterns with DNA replication‐related proteins CDC6 and MCM7. Coimmunoprecipitation experiments revealed an interaction between NAT10 and CDC6, yet the precise upstream–downstream relationships between NAT10 and CDC6/MCM7,^150^, ^151^ and how NAT10 promotes PCa progression via DNA replication, remain unclear. Moreover, the role of ac4C modification mediated by NAT10 in PCa has not been elucidated, warranting further investigation. Furthermore, the expression of NAT10 is positively correlated with the malignancy and poor prognosis of PCa. NAT10 enhances the stability of HMGA1 and KRT8 by acetylation, promoting EMT and cell cycle arrest, ultimately leads to the malignant progression of PCa.^84^

### 6.15 Renal cell carcinoma

High levels of ac4C modification and NAT10 expression were identified in clear cell renal cell carcinoma (ccRCC).^62^, ^152^ NAT10 drives tumor progression and lymphangiogenesis by facilitating the nuclear import of the Yes1‐associated transcriptional regulator (YAP1). The study also identified ankyrin repeats and zinc finger peptidyl tRNA hydrolase 1 (ANKZF1),^153^ a target of NAT10‐mediated ac4C modification, which was upregulated in ccRCC. Mechanistically, ANKZF1 interacts with YWHAE to prevent the cytoplasmic retention of YAP1,^154^ thus activating transcription factors involved in lymphangiogenesis.^155^

### 6.16 Salivary adenoid cystic carcinoma

Ge et al.’s^156^ study showed that CCAAT enhancer binding protein beta (CEBPB) was overexpressed in salivary adenoid cystic carcinoma (SACC) tissues, which correlated with lung metastasis and poor prognosis. Functional experiments have shown that CEBPB promotes the oncogenic properties of SACC by binding to the promoter region of vimentin and upregulating its expression. Overexpression of CEBPB is attributed to NAT10‐mediated ac4C modification.^156^

The upstream and downstream regulatory networks of NAT10 and the in vivo and in vitro experimental models utilized in pan‐cancer research are presented in Table 1.

**TABLE 1:** The role of NAT10 or its mediated ac4C in pan‐cancers.

Cancer type	Expression	Functional roles	Downstream	Upstream	Experiment model	References
Bladder cancer	↑	NAT10 mediates ac4C acetylation of BCL9L, SOX4, and AKT1, enhancing their mRNA stability and promoting proliferation, migration, and stemness while inhibiting apoptosis in bladder cancer.	BCL9L, SOX4, AKT1	–	Cell lines, organoids, CDX, CKO	^113^
		NAT10‐mediated ac4C acetylation of AHNAK promotes cisplatin resistance in bladder cancer by activating the AHNAK‐mediated DNA damage repair pathway.	AHNAK	p65	Cell lines, organoids, CDX	^114^
		CLIC3 interacts with NAT10 to inhibit NAT10‐mediated ac4C modification of p21 mRNA, thereby promoting bladder cancer progression.	p21	CLIC3	Cell lines, CDX	^115^
Breast cancer	↑	NAT10‐mediated acetylation of MORC2 regulates DNA damage repair.	MORC2, PAPR1	–	Cell lines	^54^, ^120^, ^157^
		Inhibition of NAT10 reverses doxorubicin resistance in breast cancer by reversing the activation of EMT.	EMT	–	Cell lines	^122^
		NAT10 mediates ac4C acetylation of drug resistance‐associated proteins MDR1 and BCRP.	MDR1, BCRP	–	Cell lines, CDX	^117^
Cervical cancer	↑	NAT10 mediates HNRNPUL1 ac4C acetylation to promote cervical cancer progression.	HNRNPUL1	–	Cell lines, CDX	^125^
		HOXC8/NAT10/FOXP1 promotes progression of cervical cancer and formation of suppressive immune microenvironment by regulating glucose metabolism.	FOXP1	HOXC8	Cell lines, CDX	^94^
		CircMAST1 competitively binds to NAT10 to repress YAP mRNA acetylation and promotes cervical cancer progression.	YAP	CircMAST1	Cell lines, CDX	^52^
Colorectal cancer	↑	NAT10 mediates KIF23 acetylation to promote colorectal cancer progression.	KIF23/Wnt/β‐catenin	–	Cell lines, CDX	^75^
		GSK‐3β‐induced subcellular redistribution of NAT10 to promote colorectal cancer progression	–	GSK‐3β	Cell lines, CDX	^128^
		NAT10‐mediated N4‐acetylation of ferroptosis suppressor protein FSP1 mRNA to promote the progression of colorectal cancer.	FSP1	–	Cell lines, CDX	^126^
		The mRNA ac4C acetylation risk score is associated with tumor immune infiltration levels, TMB, and microsatellite instability status in colon cancer.	–	–	Cell lines	^127^
Esophageal cancer	↑	Protein 2‐hydroxyisobutyrylation (Khib) modification of NAT10 is increased in metastatic esophageal cancer, facilitating its interaction with deubiquitylase USP39 and enhancing NAT10 protein stability. NAT10‐mediated ac4C acetylation of NOTCH3 promotes metastasis of esophageal cancer.	NOTCH3	USP39	Cell lines, CDX	^129^
		NAT10‐mediated ac4C acetylation of tRNA promotes EGFR translation and gefitinib resistance in esophageal cancer.	EGFR	–	Cell lines, CDX	^130^
		N4‐acetylation of lncRNA CTC‐490G23.2 promotes metastasis of esophageal cancer by increasing CD44 isoform‐selective splicing via interaction with PTBP1.	lncRNA CTC‐490G23.2	–	Cell lines, CDX	^47^
Gastric cancer	↑	NAT10‐mediated COL5A1 acetylation promotes gastric cancer metastasis.	COL5A1	–	Cell lines	^158^
		Helicobacter pylori promotes progression of gastric cancer by downregulating p53 via NAT10‐mediated ac4C acetylation of MDM2.	MDM2, p53	Hp	Cell lines, CDX	^59^
		NAT10/SEPT9/HIF‐1α positive feedback loop regulates glucose metabolism in gastric cancer.	SEPT9	HIF‐1a	Cell lines, CDX	^131^
		NAT10 mediates ac4C acetylation of SMYD2 to promote progression of gastric cancer.	SMYD2	NETs	Cell lines, CDX	^133^
		DARS‐AS1/miR‐330‐3p/NAT10 regulates progression of gastric cancer.	–	DARS‐AS1/miR‐330‐3p	Cell lines	^134^
		NAT10 modulates YTHDF1 splicing through SRF2 acetylation to drive GC progression	SRSF2, YTHDF1	–	Cell lines, Organoids, CDX	^137^
Laryngeal cancer	↑	NAT10‐mediated ac4C acetylation of FOXM1 promotes progression of laryngeal cancer.	FOXM1	–	Cell lines, CDX	^138^
Lung cancer	↑	Ac4C acetylation of mRNAs in lung adenocarcinoma affects cellular functions including protein SUMOylation and membrane transporter activities.	TFAP2A	–	–	^139^
		c‐myc upregulates the expression of NAT10, which promotes the proliferation and migration of non‐small cell lung cancer by regulating the cell cycle.	Cell cycle	c‐myc	Cell lines	^140^
Hepatocellular cancer	↑	NAT10 promotes liver cancer metastasis and epirubicin chemoresistance by activating EMT.	EMT	–	Cell lines, CDX	^141^, ^159^
		NAT10 mediates HSP90AA1 ac4C acetylation to promote endoplasmic reticulum stress‐mediated hepatocellular carcinoma metastasis and lenvatinib resistance.	HSP90AA1		Cell lines, CDX	^143^
		Downregulation of ac4C acetylation facilitates MDSCs infiltration and impairs the response to immunotherapy.	MDSCs	–	Cell lines, CDX	^142^
Osteosarcoma	↑	ac4C‐modified YTHDC1 mediates m6A methylation of PFKM and LDHA to regulate glucose metabolism and promote progression of osteosarcoma.	YTHDC1	–	Cell lines, CDX	^145^
		NAT10 mediates ac4C acetylation of FNTB to promote osteosarcoma progression	FNTB	–	Cell lines	^146^
Pancreatic cancer	↑	NAT10‐mediated ac4C acetylation of AXL promotes progression and metastasis of pancreatic cancer	AXL	–	Cell lines, CDX	^160^
Prostate cancer	↑	NAT10 promotes proliferation, invasion, and migration of prostate cancer cells through DNA replication.	CDC6	–	Cell lines	^61^
		Ac4C of HMGA1 and KRT8 promotes the progression of prostate cancer	HMGA1, KRT8	–	Cell lines, CDX	^84^
Renal cell carcinoma	↑	ANKZF1 is highly expressed in ccRCC due to NAT10‐mediated ac4C acetylation and inhibits YAP1 retention in the cytoplasm, promoting ccRCC progression and lymphangiogenesis.	ANKZF1, YAP1	–	Cell lines, CDX	^155^
Salivary adenoid cystic carcinoma	↑	NAT10 promotes proliferation, invasion, and migration of salivary adenoid cystic carcinoma.	CEBPB, Vimentin	–	Cell lines	^156^
Acute myeloid leukemia	↑	NAT10 inhibits endoplasmic reticulum stress‐related apoptosis and promotes AML progression.	ER stress, apopotosis	–	Cell lines	^112^
Malignant melanoma	↑	NAT10 promotes production of melanin and growth of melanoma.	MITF	–	Cell lines	^144^

Abbreviations: AHNAK, AHNAK nucleoprotein; ANKZF1, ankyrin repeat and zinc finger peptidyl TRNA hydrolase 1; AXL, AXL receptor tyrosine kinase; BCL9L, B‐cell lymphoma 9‐like; BCRP, breast cancer resistance protein; CEBPB, CCAAT enhancer binding protein beta; EMT, epithelial–mesenchymal transition; FNTB, the farnesyltransferase subunit beta gene; FOXM1, Forkhead box M1; FOXP1, Forkhead box P1; FSP1, ferroptosis suppressor protein 1; GLUT4, glucose transporter 4; HNRNPUL1, heterogeneous nuclear ribonucleoprotein U like 1; Khib, lysine 2‐hydroxyisobutyrylation; KHK, ketohexokinase; KIF23, kinesin family member 23; MDM2, the murine double minute 2; MDR1, breast cancer resistance protein 1; MITF, microphthalmia‐associated transcription factor; MORC2, MORC family CW‐type zinc finger 2; NOTCH3, Notch receptor 3; SEPT9, septin 9; TFAP2A, transcription factor AP‐2 alpha; YTHDC1, YTH N6‐methyladenosine RNA binding protein C1; YWHAE, tyrosine 3‐monooxygenase/tryptophan 5‐monooxygenase.

## 7 LIMITATIONS AND CHALLENGES OF CURRENT RESEARCH

Our understanding of the role of ac4C in cancers is limited. Key enzymes involved in RNA modification are generally classified into three categories based on their functions: writer, eraser, and reader. Writer catalyzes the N4‐acetylation of cytidine nucleotides, with NAT10 being the only identified writer of ac4C. Reader recognizes and binds to ac4C‐modified nucleotides, while eraser deacetylating targeted RNAs. Writer, eraser, and reader coregulate the dynamic balance of ac4C modification. Recent studies have identified the existence of potential ac4C reader NOP58 and eraser NAD‐dependent protein deacetylase sirtuin‐7 (SIRT7): ac4C‐modified RNA can interact with NOP58 and be deacetylated by SIRT7.^161^, ^162^ However, the functions of NOP58 and SIRT7 require further validation. Therefore, further studies are needed to identify a definitive eraser and reader of ac4C.

Furthermore, recent studies on ac4C in cancer were performed on cancer cell lines or patient‐derived organoids, where NAT10 is knocked down or overexpressed for in vitro validation. However, in vivo validation is limited to subcutaneous or orthotopic xenograft. To date, few studies have utilized transgenic mice models of spontaneous tumorigenesis or have NAT10 knockout mice been constructed for in vivo validation. Moreover, levels of ac4C are typically detected by dot blot assays, which are only qualitative data. Thus, there is a need to employ NAT10 knockout mouse models and develop more precise and accessible methods for detecting ac4C modifications in cancer, thereby enhancing the reliability and accuracy of these findings.

Although NAT10 and ac4C modification are promising therapeutic targets for cancer treatment, there are several limitations that should be addressed. First, no erasers or readers for ac4C have been identified. The absence of erasers and readers has hindered the development of inhibitors targeting these components. In contrast, other RNA modifications, such as m6A, may carry significant therapeutic potential because they involve multiple regulatory proteins, including readers and erasers. In the case of ac4C, however, NAT10 is the sole known regulator, limiting its versatility as a drug target. NAT10 has an inhibitor, Remodelin, effectively suppressed NAT10 expression and downregulated the level of ac4C in vitro and in vivo. However, the precise mechanism of Remodelin in not fully understood. Remodelin's binding site is not located within the catalytic domain of NAT10 responsible for ac4C modification.^163^ Furthermore, based on current literature, Remodelin has yet to undergo comprehensive clinical trials. This may be attributed to the need for further investigation into its pharmacokinetics and pharmacodynamics in humans, as well as concerns about its long‐term safety profile. In addition, it remains unclear whether Remodelin can effectively address tumor heterogeneity or whether prolonged application may induce drug resistance. This warrants further investigation.^11^, ^164^

**FIGURE 5:**
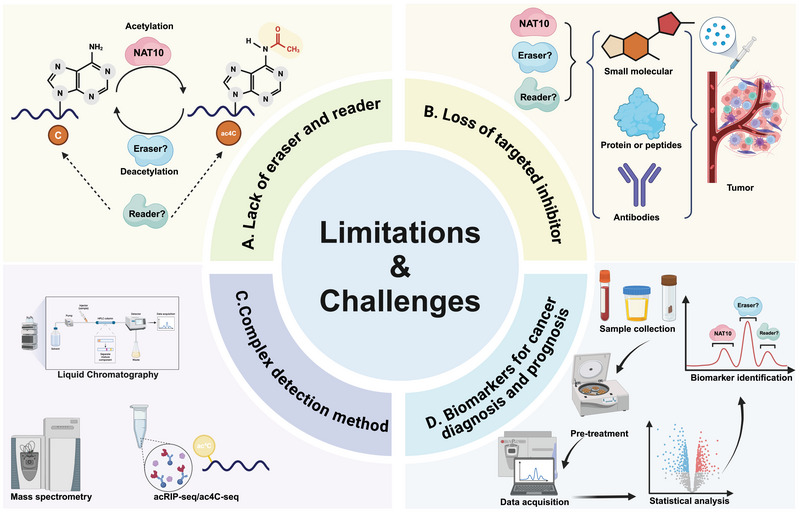
Limitations and challenges in current research of ac4C. (A) Lack of identification of reader and eraser: ac4C is a dynamic process that requires writer, eraser, and reader to regulate the ac4C modification status and function of target genes. Current studies have only identified the one and only writer NAT10, with a lack of reader and eraser studies. (B) Lack of targeted inhibitors. Targeted inhibitors for ac4C writer, eraser, and reader are required to be explored for clinical cancer therapy. (C) Lack of detection methods. Currently, the detection of ac4C relies on liquid mass spectrometry and acRIP. (D) Lack of biomarkers for diagnosis and prognosis. ac4C remains to further validate for use as a cancer biomarker. ac4C, N4‐acetylcytosine.

The significance of ac4C as a biomarker for cancer diagnosis and prognosis need to be clarified. First, compared with m6A, the abundance of ac4C modifications in human cells is relatively low, which limits it detectability. Second, the detection methods for ac4C modifications are still underdeveloped, posing challenges for its adoption in clinical settings. The varying roles and complex mechanisms of ac4C in different tumor types further restrict its applicability as a universal diagnostic marker (Figure 5).

## 8 CONCLUSION

Several studies have indicated that NAT10 is overexpressed in malignant tumors which correlates with poor prognosis. Research investigating the role of NAT10 in cancer typically adopts two primary approaches. One approach considers NAT10 as an oncogene, examining its involvement in cancer progression. NAT10 can enhance tumor cell proliferation by regulating the cell cycle, leading to G1/M phase arrest, and can facilitate tumor invasion, migration, and metastasis by suppressing apoptosis and promoting EMT.

Other approach involves investigating the NAT10‐mediated ac4C acetylation of downstream target genes, and their roles in tumorigenesis. The ac4C modification stabilizes mRNA and enhances translation, upregulating the expression of oncogenic genes and providing further insight into the cancer‐promoting functions of NAT10. Although individual genes or molecules may promote cancer progression in controlled experimental settings, their contribution to tumor advancement in a normal physiological context may be limited. However, ac4C acetylation has been shown to participate in multiple mechanisms of tumor progression, including metabolic reprogramming, immune microenvironment alternations, and drug resistance. Furthermore, carcinogenic microorganisms such as Helicobacter pylori, can drive tumor progression by modulating ac4C modifications.

Remodelin, the NAT10's inhibitor, inhibited the tumor growth by downregulating the ac4C acetylation of downstream functional genes via suppressing the expression of NAT10 in multiple cancers. In vitro and in vivo studies have demonstrated that combining Remodelin with chemotherapeutic agents or immunotherapy (e.g., PD‐1/PD‐L1 inhibitors) may be an effective strategy to control tumor progression. As a novel RNA modification, ac4C acetylation presents significant therapeutic potential, positioning NAT10 as a promising target for the treatment of multiple tumor types.^165^, ^166^, ^167^

## AUTHOR CONTRIBUTIONS

Bing Shen, Xudong Yao, Wentao Zhang, Jiang Geng, and Wei Li conceived the study. Zhuoran Gu, Libin Zou, and Xinjian Pan drafted the manuscript. Zhuoran Gu, Xinjian Pan, and Yang Yu prepared the figures and table. Yongqiang Liu and Zhijing Zhang interpreted the data. Changcheng Guo polished the article. Ji Liu collected the references. Junfeng Zhang and Shiyu Mao participated in the discussion and gave constructive comments. All authors have read and agreed to the published version of the manuscript.

## CONFLICT OF INTEREST STATEMENT

There is no conflict of interest to declare.

## ETHICS STATEMENT

Not applicable.

## Data Availability

The data that support the findings of this study are available from the corresponding author upon reasonable request.
